# Determination of the Bridging Ligand in the Active Site of Tyrosinase

**DOI:** 10.3390/molecules22111836

**Published:** 2017-10-27

**Authors:** Congming Zou, Wei Huang, Gaokun Zhao, Xiao Wan, Xiaodong Hu, Yan Jin, Junying Li, Junjun Liu

**Affiliations:** 1 Yunnan Academy of Tobacco Agricultural Sciences, 33 Yuantong Street, Kunming 650021, China; congming.zou@uky.edu (C.Z.); 13577799059@163.com (W.H.); zhaogk2002@sina.com (G.Z.); hxd20030100101@163.com (X.H.); jinyan2017ziran@163.com (Y.J.); ljy1250@163.com (J.Y.L.); 2 School of Pharmacy, Tongji Medical College, Huazhong University of Science and Technology, 13 Hangkong Road, Wuhan 430030, China; marina.wanx@gmail.com

**Keywords:** tyrosinase, active site, bridging ligand, QM/MM-PBSA, inhibitor design

## Abstract

Tyrosinase is a type-3 copper enzyme that is widely distributed in plants, fungi, insects, and mammals. Developing high potent inhibitors against tyrosinase is of great interest in diverse fields including tobacco curing, food processing, bio-insecticides development, cosmetic development, and human healthcare-related research. In the crystal structure of *Agaricus bisporus* mushroom tyrosinase, there is an oxygen atom bridging the two copper ions in the active site. It is unclear whether the identity of this bridging oxygen is a water molecule or a hydroxide anion. In the present study, we theoretically determine the identity of this critical bridging oxygen by performing first-principles hybrid quantum mechanics/molecular mechanics/Poisson-Boltzmann-surface area (QM/MM-PBSA) calculations along with a thermodynamic cycle that aim to improve the accuracy. Our results show that the binding with water molecule is energy favored and the QM/MM-optimized structure is very close to the crystal structure, whereas the binding with hydroxide anions causes the increase of energy and significant structural changes of the active site, indicating that the identity of the bridging oxygen must be a water molecule rather than a hydroxide anion. The different binding behavior between water and hydroxide anions may explain why molecules with a carboxyl group or too many negative charges have lower inhibitory activity. In light of this, the design of high potent active inhibitors against tyrosinase should satisfy both the affinity to the copper ions and the charge neutrality of the entire molecule.

## 1. Introduction

Tyrosinase (Enzyme Commission, EC 1.14.18.1) is a type-3 copper enzyme that is widely distributed in plants, fungi, insects, and mammals [1,2,3]. It catalyzes both the hydroxylation of monophenols and the oxidation of *o*-diphenols, and thus is directly associated with plant browning [4], cuticle formation [3], and skin pigmentation [1,5]. Tyrosinase is also involved in Parkinson’s disease, due to its ability to oxidize dopamine to form melanin in the brain [6]. As a result, considerable efforts have been invested in developing safe and high-potential tyrosinase inhibitors in diverse fields including tobacco curing [7], food processing [8], bio-insecticides development [3], cosmetic development [9], and human healthcare-related research [10,11].

**Figure 1:**
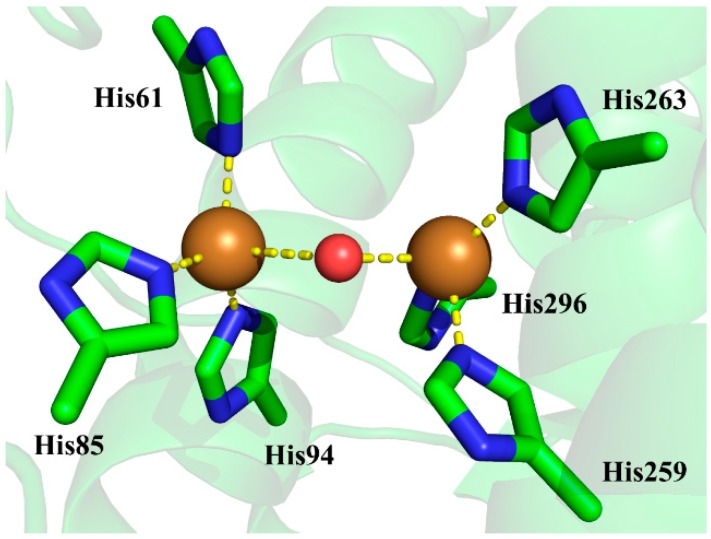
The active site of the crystal structure of *Agaricus bisporus* mushroom tyrosinase (Protein Data Bank, PDB ID 2Y9W). The side chains of histidine residues are rendered as sticks, while copper ions and bridging oxygen are rendered as spheres. The mode system in the present study is defined by the atoms in stick and sphere representations. The carbon, nitrogen, oxygen, and copper atoms are colored with green, blue, red, and brown, respectively.

The active site of tyrosinase contains six conserved histidine residues coordinating two copper ions [12], which are critical for both catalytic activities of tyrosinase. Shown in Figure 1 is the active site of the crystal structure of *Agaricus bisporus* mushroom tyrosinase. As we can see, an oxygen was determined at a location bridging the two copper ions, and it is uncertain whether this bridging oxygen is a water molecule or a hydroxide anion since hydrogen atoms cannot be determined by X-ray diffraction techniques [12].

**Figure 2:**
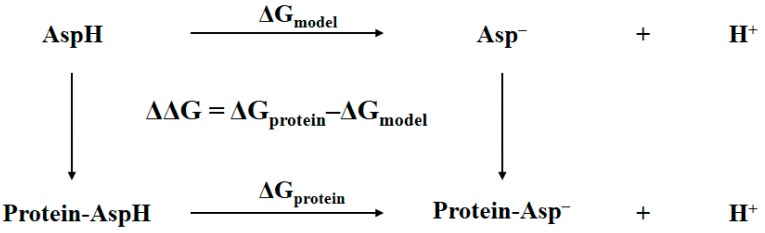
Thermodynamic cycle for determining the p*K*_a_ of aspartic residue in protein. The double free energy difference ΔΔG is for p*K*_a_ shifts analysis.

In general, the determination of the identity of the bridging oxygen can be achieved by comparing the Gibbs free energy of the protein with bridging water to that of the protein with bridging hydroxide anion plus proton in aqueous solution. The accuracy of this comparison can be improved by using a thermodynamic cycle where a model system is needed [13,14]. Shown in Figure 2 is an example of such a thermodynamic cycle that had been successfully used in p*K*_a_ prediction or protonation state determination for an aspartic residue in protein [13]. The ΔG_model_ is the free energy difference of the model system, which is the aspartic acid in aqueous solution, changing from the protonated state to the deprotonated state. The ΔG_protein_ is the free energy difference of the corresponding aspartic residue in protein changing from the protonated state to the deprotonated state. The double free energy difference ΔΔG is then equivalent to the p*K*_a_ shifts of aspartic acid migrating from aqueous solution to protein, i.e., p*K*_a,protein_ − p*K*_a,model_ = ΔΔG/RT*ln*(10). This equation can be re-written as follows:

(1) $$\Delta G_{\mathrm{protein}(\mathrm{expt})}=\Delta G_{\mathrm{protein}(\mathrm{calc})}-\Delta G_{\mathrm{model}(\mathrm{calc})}+\Delta G_{\mathrm{model}(\mathrm{expt})}$$

The ΔG_protein(expt)_ can be used to determine the p*K*_a,protein_ of aspartic residue in protein and to tell which protonation state is more favorable. As long as the model system has the experimentally determined p*K*_a,model_ available and is structurally very close to its counterpart in protein, this thermodynamic cycle along with Equation (1) is highly efficient in p*K*_a_ prediction or protonation state determination, because the systematic error caused by the theoretical method can be largely eliminated. In addition, the absolute free energy of the proton in aqueous solution is no longer necessary, and therefore the calculations of ΔG_protein(calc)_ and ΔG_model(calc)_ are basically the free energy difference between the deprotonated and protonated states of the molecules. These two values could be estimated by the so-called QM/MM-PBSA method, in which the QM/MM refers to the hybrid quantum mechanical/molecular mechanics and PBSA refers to the solvation energy evaluated by solving Poisson–Boltzmann (PB) equation, and by estimating the nonpolar contribution in terms of the change in solvent-accessible surface area (SA). The QM/MM-PBSA method has been successfully applied to many protein systems, including reactions in proteins [15], binding mode analysis [16,17], inhibition constant evaluation [18], and protonation state determination [19]. By incorporating the abovementioned thermodynamic cycle, it should produce a reliable result for the values of ΔG_protein(calc)_ and ΔG_model(calc)_.

To determine the identity of the critical bridging oxygen in the active site of tyrosinase, we may employ the QM/MM-PBSA method, incorporating a similar thermodynamic cycle as that in Figure 2. The most appropriate model system should be a small cluster representing the active site of tyrosinase, and practically could be the atoms in the stick and sphere representations shown in Figure 1. It contains the side chains of six histidine residues coordinating two copper ions, in which the two copper ions are connected by a bridging water or a bridging hydroxide anion. Unfortunately, this model system does not have the experimental p*K*_a_ value available, causing the lack of ΔG_model(expt)_, and consequently prevents the use of Equation (1) to estimate ΔG_protein(expt)_. However, it is possible to accurately estimate ΔG_model(expt)_ by using first-principles quantum mechanical calculations with the continuum solvation model. The pure continuum solvation model is well-suited to describe the long-range electrostatic interactions between solute and the bulk solvent, but only partially considers the short-range electrostatic interactions, e.g., the electrostatics between the solute and first solvation shell [20,21,22]. As a result, the inclusion of water molecules in the solvation shell around the solute to form a solute-water cluster can largely improve the calculation accuracy for ions where solvation effects are significant [23,24]. For example, the absolute hydration free energy of the proton was determined in such a way by including the water clusters in the first and second solvation shells [25]. In addition, a recent study showed that the average error for p*K*_a_ prediction on molecules with hydroxyl and hydroperoxyl functional groups was about 0.02 p*K*_a_ units by employing the SMD solvation model [26] at the B3LYP/6-311++G** level, in which the continuum/cluster model was used with three explicit water molecules in the first solvation shell [27]. Accordingly, the ΔG_model(expt)_ for the model systems here could be calculated by using the same method.

In this study, we theoretically determine the identity of the critical bridging oxygen by performing first-principles QM/MM-PBSA calculations along with a thermodynamic cycle that aim to improve the accuracy. By analyzing the QM/MM-optimized geometries, the keys to design high potent active inhibitors against tyrosinase are discussed.

## 2. Methods

### 2.1. Thermodynamic Cycle

**Figure 3:**
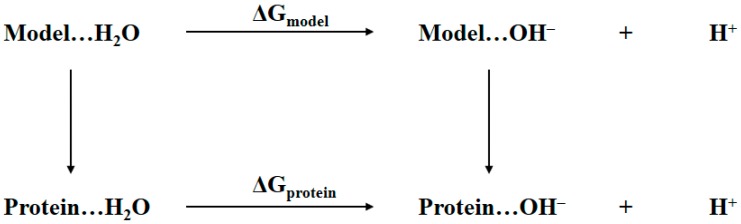
Thermodynamic cycle for determining the identity of bridging oxygen, i.e., water or hydroxide anion, in the active site of tyrosinase.

The determination of the bridging ligand in the active site of tyrosinase to be water or hydroxide anion is essentially the same as determining the protonation state of aspartic residue in protein shown in Figure 2. Thus, a similar thermodynamic cycle is built in Figure 3. The notations Protein…H_2_O and Protein…OH^−^ refer to the two tyrosinase systems of which the bridging ligand is water or hydroxide anion, respectively. The Model refers to a cluster model system that is structurally as close as possible to the active site of tyrosinase, namely the atoms rendered in stick and sphere representations in Figure 1. The ΔG_protein_ and ΔG_model_ are the Gibbs free energy changes from bridging water to bridging hydroxide anion for tyrosinase and the model systems, respectively. In the present study, these two values are obtained by using the QM/MM-PBSA calculations. Considering the fact that the p*K*_a_ for the model system is determined by first-principles QM calculations, the notations of terms in Equation (1) shall be revised in a more appropriate form:

(2) $$\Delta G=\Delta G_{\mathrm{protein}\left(\mathrm{QM}/\mathrm{MM}-\mathrm{PBSA}\right)}-\Delta G_{\mathrm{model}\left(\mathrm{QM}/\mathrm{MM}-\mathrm{PBSA}\right)}+\Delta G_{\mathrm{model}(\mathrm{QM})}$$

in which the value of ΔG can be used to determine the identity of the bridging ligand.

### 2.2. QM/MM-PBSA Calculation

In the QM/MM-PBSA method, the free energy is calculated by using the following equation:

G = E_QM/MM_ + G_PBSA_ − TS_QM/MM_ (3)

where E_QM/MM_ is the QM/MM potential energy; G_PBSA_ is the solvation energy that consists of the polar solvation energy estimated by solving the Poisson-Boltzmann equation and the nonpolar solvation energy estimated from the solvent-accessible surface area of the molecule; T is the room temperature; and S_QM/MM_ is the entropy of the molecule, which could be estimated as:

S_QM/MM_ = S_QM_ + S_MM_ + S_QM-MM_ (4)

S_QM_ and S_MM_ are the entropy terms of QM and MM systems, respectively. S_QM-MM_ is the coupling term between QM and MM systems.

The crystal structure 2Y9W was used for the preparation of the initial structure for QM/MM calculations. The QM system is depicted as the atoms that are rendered as sticks and spheres in Figure 1. It consists of the two copper ions, the side chains of histidine residues 61, 85, 94, 259, 263, and 296 that form around the copper ions, and the bridging ligand which is a water molecule or a hydroxide anion. The rest of the atoms are defined as the MM system. The QM/MM interface was described by the link-atom method where the boundary atoms are capped by hydrogen atoms. [28]. The QM/MM calculations were carried out by using an interface program implemented in Amber16 [29] at the B3LYP/6-31+G*:AMBER level, in which the QM system was described at the B3LYP/6-31+G* level of theory by using Gaussian 09 program [30] and the MM system was described with the Amber14SB force field by using the sander module in Amber16. In all QM/MM calculations, atoms within 15 Å of the two copper ions were allowed to move while all the other atoms outside this range were kept frozen. The geometry optimizations were performed by using the Limited-memory Broyden-Fletcher-Goldfarb-Shanno (LBFGS) algorithm [31] implemented in the sander program. The convergence criterion is the root-mean-square (RMS) deviation of the energy gradient being less than 0.01 kcal·mol^−1^·Å^−1^. At the convergence of the geometry optimization, the RESP (restrained electrostatic potential) charges [32] of the QM system along with the MM charges obtained from the QM/MM calculations were applied in the PBSA calculations by using the PBSA program in Amber16.

The QM/MM and PBSA calculations generate the first and second terms in Equation (3). For the entropy term that is described with details in Equation (4), we ignored the term S_MM_ because it is unlikely that the entropy of the protein is significantly different for the two states. Likewise, the contributions from bonded and van der Waals QM-MM interactions to the term S_QM-MM_ were also ignored, because S_QM-MM_ majorly comprises the contribution from QM-MM electrostatic interactions. Technically, the entropy term was calculated by using Gaussian 09 program with normal mode analysis of harmonic frequencies for the QM system, in which MM point charges were taken as background charges. The QM/MM-PBSA calculations were performed on both protein and model systems in order to cancel the systematic errors from the theoretical method. Since the model system is defined exactly the same as that for the QM system, the calculations for the model system are basically the QM/MM-PBSA calculations on an isolated QM system that does not have MM background charges.

### 2.3. QM Calculation for pK_a_ Determination

The p*K*_a_ could be accurately determined by first-principles QM calculations employing the continuum/cluster model. For example, it was found that the SMD solvation method incorporating three explicit water molecules in the first solvation shell produced accurate p*K*_a_ for molecules with hydroxyl and hydroperoxyl functional groups [27]. In the present study, the oxygen of the bridging ligand binds with the two copper ions, leaving the hydrogens of the bridging ligand pointing towards the outside of the active site. Thus, there are two water molecules in the first solvation shell for bridging water, and one water molecule in the first solvation shell for bridging hydroxide anion. In order for the computed energies to be reliable as well as comparable, the water molecules in the first and second solvation shells are explicitly included, resulting in three explicit water molecules for both bridging water and bridging hydroxide anion states of model system. We carried out constrained molecular dynamics (MD) simulations on the model system to obtain the three explicit water molecules at reasonable locations. Since the model system is defined as the same as that for the QM system, the RESP charges for the QM system were used here for the model system in the constrained MD simulation. The model system was solvated in a rectangular box of TIP3P water molecules with a minimum solute wall distance of 8 Å. Prior to MD simulation, the energy minimization was carried out to remove possible bad contacts. Then, the equilibration was performed for 40 ps with the temperature gradually increasing from 10 K to 300 K, followed by a 50-ps simulation at 300 K in NPT ensemble, i.e., isothermal–isobaric ensemble. The time step in the MD simulation was 2 fs. It is worth noting that the purpose of the MD simulation here was to obtain the three explicit water molecules at reasonable locations, and the geometry of the model system constructed from the crystal structure should not be altered by the molecular force field, in case the molecular force field may not be appropriate for describing the bi-nuclear copper system. Therefore, in the energy-minimization and the MD simulation, only water molecules and the hydrogen atoms were allowed to move, and all remaining atoms were kept fixed at their crystal structure positions. The three water molecules that had closest contacts with the bridging ligand were selected from the last snapshot of the MD simulations. After that, the two states of the model system, i.e., the bridging ligand as water molecule or hydroxide anion, respectively, were subjected to the geometry optimizations at the B3LYP/6-31+G* level of theory with the SMD solvation method by using Gaussian 09 program. The optimized geometries were verified by harmonic normal mode calculations. The energies of the two states were further refined by single-point calculations at the B3LYP/6-311++G** level of theory. The free energy of proton in aqueous solution was calculated as:

(5) $$G_{\mathrm{aq},H^{+}}=G_{g\mathrm{as},H^{+}}^{1\mathrm{atm}}+\Delta G^{1\mathrm{atm}\to 1M}+\Delta G_{\mathrm{solv},H^{+}}^{1M}$$

1 M and 1atm refer to the standard states for the aqueous solution and gas phase, respectively. G_gas,H^+^_ is the gas-phase free energy of the proton with the value of −6.28 kcal/mol at the 1 atm standard state and 298.15 K [27]. ΔG^1atm→1 M^ is the free energy change from the standard state of 1 atm to 1 M, of which the value is 1.89 kcal/mol [33]. ΔG_solv,H^+^_ is the hydration energy of proton at the 1 M standard state and 298.15 K, of which the value is −265.9 kcal/mol [25,33]. Therefore, the free energy of the proton in aqueous solution at the standard state of 1 M and 298.15 K is −270.3 kcal/mol. Finally, the free energy difference of the model system changing from bridging water to bridging hydroxide anion is calculated by Equation (6), which can be converted to p*K*_a_ by Equation (7):

ΔG_model(QM)_ = G_model(hydroxide anion)_ + G_aq,H^+^_ − G_model(water)_ (6)

(7) $$pK_{a}=\frac{\Delta G_{\mathrm{model}\left(\mathrm{QM}\right)}}{\mathrm{RTln}10}$$

## 3. Results and Discussion

### 3.1. Charge States of Copper Ions

**Figure 4:**
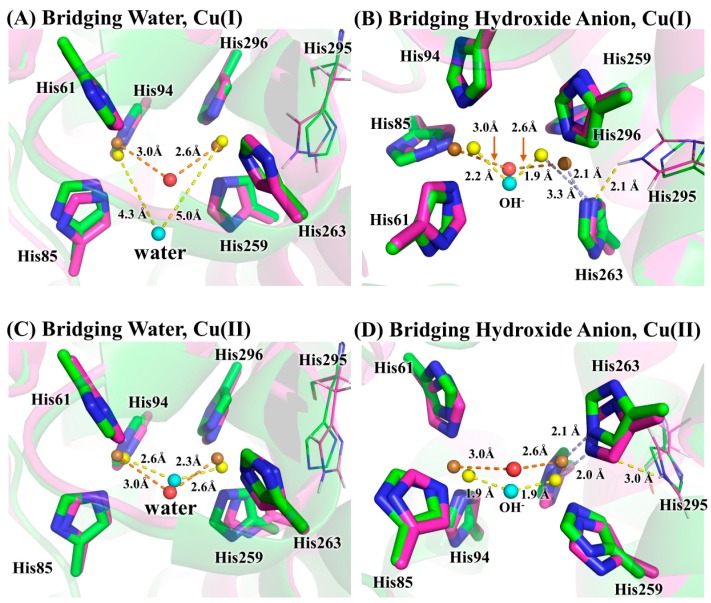
The comparison of four possible QM/MM-optimized active site structures to that of crystal structure 2Y9W. The bridging ligand is water in (**A**,**C**); and is hydroxide anion in (**B**,**D**). The copper ions are Cu(I) in (**A**,**B**); and are Cu(II) in (**C**,**D**). For crystal structure 2Y9W, the carbon, nitrogen, oxygen, and copper atoms are colored with green, blue, red, and brown, respectively, whereas for the QM/MM-optimized structures these atoms are colored with magenta, blue, cyan, and yellow, respectively. For clarity, the copper ions and the bridging oxygen are scaled to small spheres.

The two copper ions in tyrosinase may take two possible charge states [11], i.e., the +1 formal charge corresponding to the singlet state, and the +2 formal charge corresponding to the triplet state. The two charge states of copper ions are denoted by Cu(I) and Cu(II), respectively. By combining these two charge states with the two possibilities of bridging ligands, we have four possible structures for the active site of tyrosinase, which were subjected to QM/MM optimizations at the B3LYP/6-31+G*:AMBER level of theory. Shown in Figure 4 are the comparisons of the four QM/MM-optimized structures to the crystal structure 2Y9W. We can see from Figure 4A that the bridging water molecule is unable to bind with the two Cu(I) ions. If the bridging ligand is hydroxide anion, as shown in Figure 4B, the bridging ligand may keep the binding with two Cu(I) ions. However, the distance between the right side copper ion and the epsilon nitrogen of His263 changes from 2.1 Å in the crystal structure to 3.3 Å in the QM/MM-optimized structure, indicating that the coordination of copper ion to His263 is lost. This is further confirmed by the fact that His295 forms a hydrogen bond with His263. Clearly, no matter what the bridging ligand is, the coordination of copper ions cannot be maintained when the two copper ions are at the singlet state, suggesting that the copper ions in the crystal structure 2Y9W are not Cu(I). On the other hand, as we can see from Figure 4C,D, the coordination of copper ions are maintained on both the bridging water and bridging hydroxide anion systems when the copper ions are at the triplet state. In addition, the hydrogen bond formed between side chains of His263 and His295 in Figure 4B, which is an indication that the coordination of the right side Cu(I) is disrupted, is not observed in Figure 4D, where copper ions are Cu(II). As a result, the copper in crystal structure 2Y9W should be Cu(II).

### 3.2. Determination of Bridging Ligand Identity

**Figure 5:**
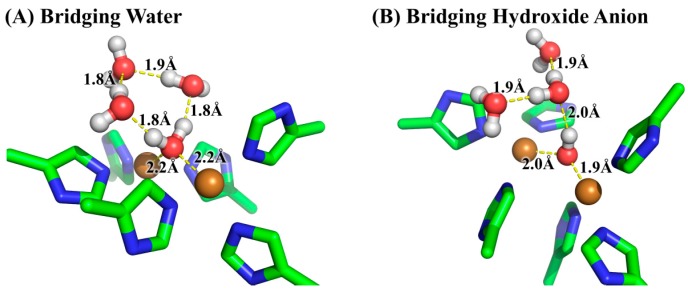
The optimized geometries for the two states of the model system at the B3LYP/6-31+G* level of theory. (**A**) The bridging ligand is water; (**B**) The bridging ligand is hydroxide anion. Three water molecules in the first and second solvation shells of the bridging ligand were added explicitly for reliable solvation effect evaluation as well as for obtaining comparable energies.

**Figure 6:**
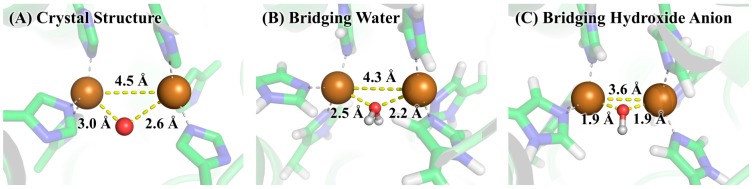
The active site of tyrosinase in (**A**) crystal structure 2Y9W; (**B**) QM/MM-optimized geometry with bridging water; and (**C**) QM/MM-optimized geometry with bridging hydroxide anion. The geometry optimizations were performed by using the QM/MM method at the B3LYP/6-31+G*: AMBER level of theory.

The free energy difference for the model system changing from the bridging water state to the bridging hydroxide state was calculated by the SMD solvation model with three explicit water molecules in the first and second solvation shells at the B3LYP/6-311++G**//B3LYP/6-31+G* level of theory. The optimized geometries of the two states of the model system are given in Figure 5. As can be seen from Table 1, the calculated ΔG_model(QM)_ is 2.8 kcal/mol and the corresponding p*K*_a_ is 2.1, indicating that the bridging ligand in the model system is hydroxide anion at a neutral pH condition. To determine the identity of the bridging oxygen in protein, we still need the values of ΔG_protein(QM/MM-PBSA)_ and ΔG_model(QM/MM-PBSA)_ in terms of Equation (2). For this purpose, QM/MM-PBSA calculations were performed on both the protein and model systems at the B3LYP/6-311++G**//B3LYP/6-31+G* level of theory. Given in Table 2 are the calculated results for these two terms, whose values are 295.3 and 260.9 kcal/mol, respectively. According to Equation (2), the ΔG for determining the bridging ligand identity should be 37.1 kcal/mol, indicating that the identity of the bridging oxygen in protein must be a water molecule rather than a hydroxide anion. This conclusion can be further confirmed by the comparison between the structures in crystal structure and those in the QM/MM-optimized geometries, as shown in Figure 6. In crystal structure 2Y9W, the distance between the two copper ions is 4.5 Å. This distance is barely changed with the bridging ligand being a water molecule. However, if the bridging ligand is a hydroxide anion, the distance between the copper ions dramatically changes to 3.6 Å. In addition, the distances between the ions and the bridging oxygen are slightly altered from 3.0 and 2.6 Å to 2.6 and 2.3 Å, respectively, if the bridging ligand is a water molecule, whereas these two distances both significantly decrease to 1.9 Å if the bridging ligand is a hydroxide anion. Clearly, the QM/MM-optimized geometry of the active site with the bridging water is very close to that in the crystal structure, which is also consistent with the result from our QM/MM-PBSA free energy evaluations. As a result, the identity of the bridging ligand in the active site of tyrosinase is determined to be a water molecule.

### 3.3. Implication for the Design of Tyrosinase Inhibitors

A recent virtual screening study [34] showed that a tetrazole group was indispensable for the inhibitory activity against tyrosinase and one of the molecules with a tetrazole group exhibited the strongest inhibitory activity, although several other molecules with a carboxyl group were also subjected to the activity tests. This is a very interesting discovery, since the positive charged copper ions are also the binding sites to inhibitors and are supposed to have higher affinity to the negatively charged carboxyl group than to the neutral tetrazol group. As shown in Figure 6, after the bridging water in Figure 6B lost a hydrogen to become the hydroxide anion in Figure 6C, the bridging oxygen moves much closer to the copper ions, indicating much stronger electrostatic interactions between copper ions and hydroxide anion. This strong interaction also makes the two copper ions move dramatically closer to each other, and consequently generates an active site that is distinct from the crystal structure, which turns out to be energy unfavorable. Obviously, although molecules with negative charges may bind geometrically tighter with the copper ions, it causes the increase of the overall energy and thus is not favorable for the binding. Therefore, molecules with a carboxyl group should have lower binding affinity when compared to their neutral counterparts. Another example is of phthalic acid (PA) and cinnamic acid (CA), whose charges are −2 and −1 at pH 7 condition, respectively. Kinetic data on these two molecules show that PA has much lower inhibitory activity against tyrosinase than that of CA [35], suggesting that too many negative charges decrease the inhibitory activity. As a result, to design highly potent active inhibitors against tyrosinase, the molecule should have affinity to the copper ions as well as a neutral charge.

## 4. Conclusions

The present results obtained from QM/MM-PBSA calculations strongly support the conclusion that the bridging ligand in the active site of the crystal structure of *Agaricus bisporus* mushroom tyrosinase is a water molecule rather than a hydroxide anion. Although hydroxide anion binds geometrically tighter with the copper ions, it significantly changes the geometry of the active site of tyrosinase and causes an increase of the overall energy. The different binding behavior between water and hydroxide anion may explain why molecules with a carboxyl group or too many negative charges have lower inhibitory activity. In light of this, the design of highly potent active inhibitors against tyrosinase should satisfy both the affinity to the copper ions and the charge neutrality of the entire molecule.

## Figures and Tables

**Table 1:** Calculated value of ΔG_model(QM)_^a^.

	E_QM_^b^	dG_QM_^c^	G_QM_^d^
water	−3,250,650.3	389.7	−3,250,260.6
hydroxide anion	−3,250,365.7	378.2	−3,249,985.3
proton	N/A	N/A	−270.3 ^e^

^a^ ΔGmodel_(QM)_ = G_model(hydroxide anion)_ + G_aq,H^+^_ − G_model(water)_ = 2.8 kcal/mol. All energies are in the unit of kcal/mol and are calculated at the B3LYP/6-311++G**//B3LYP/6-31+G* level of theory; ^b^ E_QM_ is the electronic energy; ^c^ dG_QM_ is the thermal correction to Gibbs free energy; ^d^ G_QM_ is the Gibbs free energy of each species and is calculated as the sum of E_QM_ and dG_QM_; ^e^ the Gibbs free energy of the proton in aqueous solution is calculated according to Equation (5).

**Table 2:** Calculated values of ΔG_protein(QM/MM-PBSA)_ and ΔG_model(QM/MM-PBSA)_^a^.

		E_QM/MM_^b^	dG_QM/MM_^c^	E_PBSA_^d^	G_QM/MM-PBSA_^e^	ΔG_(QM/MM-PBSA)_^f^
protein	water	−3,111,906.7	354.9	−4967.8	−3,116,519.6	295.3
	hydroxide anion	−3,111,396.4	357.4	−5185.3	−3,116,224.3	
model	water	−3,105,710.4	350.1	−463.6	−3,105,823.9	260.9
	hydroxide anion	−3,105,646.3	344.3	−260.9	−3,105,563.0	

^a^ all energies are in the unit of kcal/mol and are calculated at the B3LYP/6-31+G*:AMBER level of theory; ^b^ E_QM/MM_ is the electronic energy; ^c^ dG_QM_ is the thermal correction to Gibbs free energy for the QM system with embed MM background charges; ^d^ E_PBSA_ is the solvation energy calculated by the PBSA method; ^e^ G_QM/MM-PBSA_ is the Gibbs free energy of each species and is calculated as the sum of E_QM/MM_, dG_QM/MM_, and E_PBSA_; ^f^ ΔG_(QM/MM-PBSA)_ is calculated as G_hydroxide anion(QM/MM-PBSA)_ − G_water(QM/MM-PBSA)_.
